# Parathyroid Adenoma Presenting as a Brown Tumour of the Mandible

**DOI:** 10.1155/2012/574316

**Published:** 2012-11-07

**Authors:** Kavit Amin, Bertram Fu, Carmelo Barbaccia

**Affiliations:** Head and Neck Surgery, Medway Maritime Hospital, Kent ME7 5NY, UK

## Abstract

*Background*. Parathyroid adenoma is the commonest cause of primary hypercalcaemia and usually presents with symptoms/signs of hypercalcaemia. This paper highlights an unusual presentation. *Case Report*. A 27-year-old female presented with a painful left mandibular swelling, suspicious of neoplasia. A computed tomography (CT) guided biopsy was performed. Based on the histology result, serum calcium was carried out, confirming hypercalcaemia. A left inferior parathyroid adenoma was subsequently removed. CT mandible showed extensive erosive lesions at the left 2nd/3rd inferior molar roots with protrusion to adjacent soft tissues. USS revealed a hypoechoic lesion on the left inferior parathyroid gland. Sestamibi scan showed a focus of MIBI uptake and retention at the inferior aspect of the left thyroid lobe. *Conclusion*. This case highlights the importance of a thorough history and examination. Clinicians should always bear in mind atypical presentations of parathyroid adenomas, with the need to exclude this differential in the presence of hypercalcaemia.

## 1. Parathyroid Adenoma Presenting as a Brown Tumour of the Mandible

Parathyroid adenoma is the commonest cause of primary hypercalcaemia. It usually presents with symptoms of hypercalcaemia as a result of the increased secretion of parathyroid hormone (PTH) by the adenoma. PTH has multiple receptors, which it acts upon in order to raise blood calcium concentrations. It acts at the distal renal tubules to reabsorb calcium. Furthermore, it also allows bone resorption by indirectly stimulating osteoclastic activity and enhances absorption of calcium in the intestine by increasing the production of activated vitamin D.

When primary hyperparathyroidism is associated with bone involvement it is termed a “brown tumour”, otherwise known as osteitis fibrosa cystica. Patients with brown tumours most often present with symptoms of hypercalcaemia. Bone involvement is often regarded as a late manifestation of primary hyperparathyroidism. These tumours have also been found to occur with parathyroid carcinoma, but are a much rarer presentation [1]. Brown tumours are so called after their “brown” appearance as a result of haemosiderin and fibroblastic tissue penetrating into the gaps created in the bone matrix by the increased osteoclastic activity. This increased osteoclastic activity causes expansion of the bone beyond its normal contours. This can involve the periosteum resulting in bone pain, a presentation that can be misinterpreted as bone expansion. Brown tumours can be a presentation of both primary and secondary hyperparathyroidism. They have been reported to occur in 4.5% of patients with primary disease, and 1.5% of patients with secondary disease [2].

## 2. Clinical Report

A 27-year-old Caucasian lady presented to the Oral-Maxillofacial Department with a swelling over the left body of mandible. She noticed the swelling four weeks prior to presentation, which had been gradually increasing in size. She reported no recent dental problem or dental surgery or trauma to the left side of her jaw. The patient did not have any facial numbness but described a “bruised like” sensation over the left angle of her mandible, which was tender to touch. She was otherwise systemically well, and reported no weight loss, fevers, or rigors. The patient had no other significant medical history and was not on any regular medications, was a nonsmoker, and consumed minimal quantities of alcohol. 

On physical examination she was apyretic and systemically well, with no cervical lymphadenopathy. The left cheek was visibly more swollen than the contralateral side, which exhibited no evidence of infection on clinical assessment. Intraorally, there was expansion of the left posterior body of mandible inferior to the three left lower molars encroaching onto the horizontal ramus. The swelling was noted to be firm, but upon palpation did not appear to be of bony origin. Maximal mouth opening was normal for the patient. The floor of mouth was soft on palpation. Initial differential diagnoses included ameloblastoma, traumatic bone cyst, and an odontogenic cyst. 

**Figure 1:**
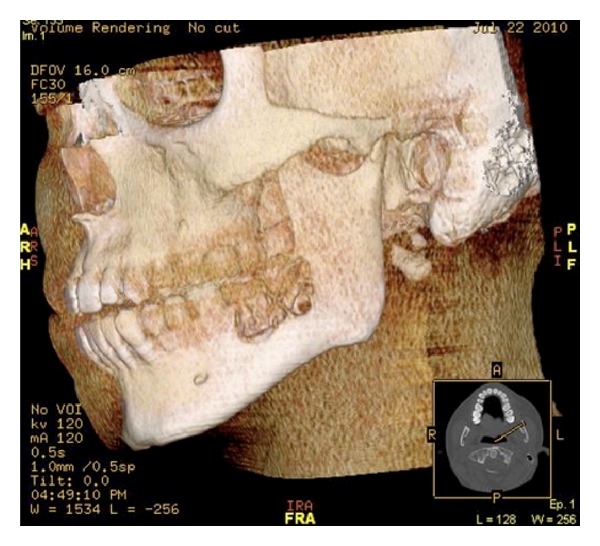
3D reconstruction of left body/ramus mandibular brown tumour.

**Figure 2:**
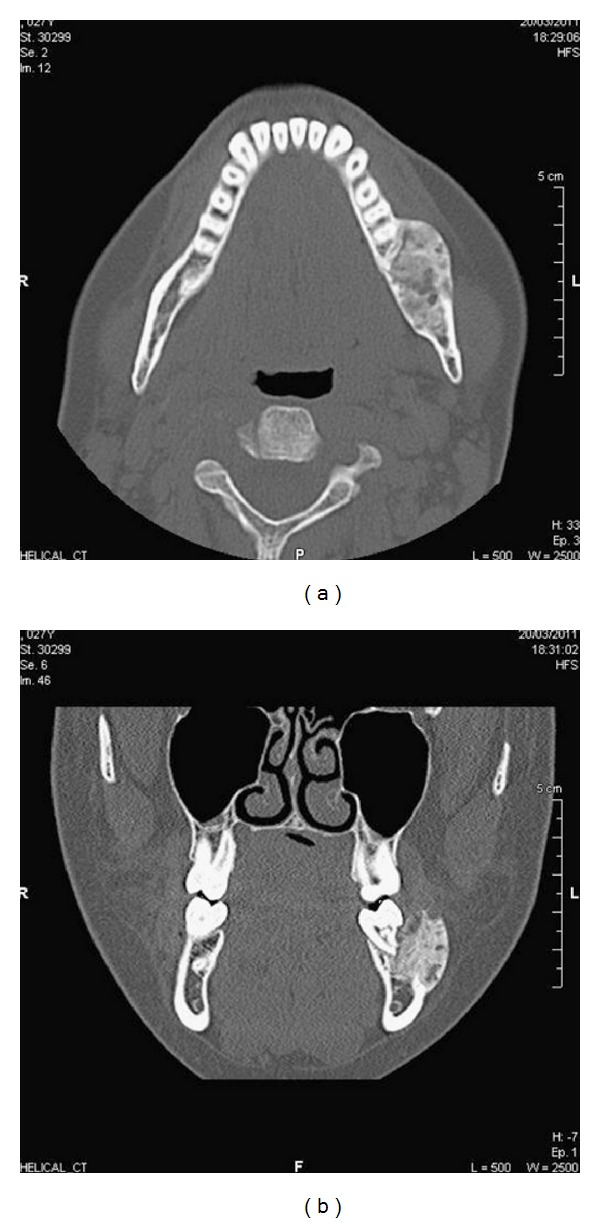
(a) Axial CT of the mandible showing the brown tumour as a mass of the left hemimandible. (b) Coronal CT scan of the brown tumour with well-defined radiolucent osteolytic lesions encircling the roots of teeth.

Computed tomography (CT) mandible revealed extensive erosive lesions at the contours of the roots of the left second and third lower molars with frank disruption of the medial and lateral cortices (Figure 1). Erosive lesions in the horizontal ramus of the left hemimandible surrounding the roots of the second and third inferior molars were also identified. Protrusion and extension to the adjacent soft tissues were noted. Anteriorly to the left masseteric area the lesion measured approximately 1.6 × 2.5 × 2.5 cm (Figure 2). There was preservation of the left inferior dental nerve on the left side with no other nerve involvement evident. 

An urgent biopsy of the lesion was undertaken to rule out the clinical suspicion of a malignant neoplasm. Histopathology revealed cellular, haemorrhagic fibrous connective tissue packed with multinucleate giant cells, with possible differentials of giant cell granuloma or hypercalcaemia. Serum biochemistry revealed a serum calcium level of 3.16 mmol/L (normal range 2.2–2.6 mmol/L), PTH level of 516 ng/L (normal range 10–65 ng/L) with alkaline phosphatase, renal and liver profiles within normal limits. 

An urgent referral to endocrinology colleagues was made with a decision to pursue for an ultrasound of the neck to investigate for parathyroid pathology. Ultrasound (US) revealed a large hypoechoic lesion on the left inferior parathyroid gland measuring 2.64 × 2.41 cm. Sestamibi scan of the parathyroids showed a large focus of MIBI (methoxyisobutylisonitrile) uptake at the inferior aspect of the left thyroid lobe. On SPECT (single photon emission computed tomography), confirmation of a parathyroid adenoma was confirmed as the cause of the patient's hypercalcaemia. She was commenced on nasal calcitonin 200 IU and referred to ENT colleagues. With concordant US and Sestamibi results, the patient underwent a selective left inferior parathyroidectomy. 

Histology revealed a circumscribed tumour composed of nests and islands of mainly chief cells. Tumour cells were noted in the capsule focally without evidence of mitoses. Histopathology confirmed the resected lesion to be a parathyroid adenoma. 

The patient recovered well postoperatively and serum calcium level normalised within a week. At the nine-month follow-up clinic review, blood tests revealed normal range calcium and PTH levels. Clinical examination showed a complete resolution of the jaw tumour with no residual swelling or overlying skin or mucosal skin changes.

## 3. Discussion

Diagnosis of brown tumours is merely presumptive, as the differential diagnosis is based on histological examination. Radiological and laboratory data are required for definitive diagnosis. They usually arise at the base of the skull, orbits, paranasal sinuses, spinal column, tibia, humerus and clavicles, and the jaws of young individuals. However, rare presentations have been found in the maxilla [4].

Our patient exhibited none of the classical symptoms of hypercalcaemia, usually referred simplistically as symptoms causing “bony pain/bone fractures, renal stones, abdominal groans, and psychic moans.” There was no history of paraesthesiae, headaches, recent fractures, constipation, or polyuria/polydipsia, symptoms usually associated with hypercalcaemia. Most patients with hypercalcaemia are asymptomatic, and are usually identified as part of routine investigations [3]. 

Initial imaging revealed well-defined radiolucent osteolytic lesions encircling the roots of teeth. Management revolves primarily in reducing circulating endocrine hormone (PTH). Medical management is an option but may be insufficient in most cases. In late diagnoses and those that do not respond to medical treatment, parathyroidectomy is the treatment of choice [4]. Parathyroidectomy is well recognized to cause resolution of lytic lesions within the jaw, without resorting to surgical intervention. A study aimed to evaluate the management of bony lesions after parathyroidectomy had been performed. In a series of 22 patients (15 mandibular in origin) with primary hyperparathyroidism with confirmed brown tumours, 21 patients has their serum calcium normalized. Furthermore, all the tumours exhibited spontaneous regression. 18 cases regressed completely, with a mean period of 10 months. Two further cases regressed completely after 2 years, and the last 2 were lost to follow-up [5].

Bisphosphonate use in patients with primary hyperparathyroidism and brown tumours has been investigated. Hypocalcaemia is a common problem post parathyroidectomy. However, in some cases this can be more prolonged despite normal or elevated PTH. This is termed “hungry bone syndrome” and seen in those with bone disease preoperatively secondary to chronic increases in bone resorption from elevated PTH. The use of bisphosphonates prior to parathyroidectomy appears to reduce bone remodelling and prevents attenuation of hypocalcaemia secondary to hungry bone syndrome post-operatively [6]. Furthermore, calcitonin can also be used to block the effects of PTH with minimal side effects reported [7].

## 4. Conclusion

Brown's tumours are less often seen in current times with patients managed long before the onset of tumour development. They cause significant morbidity and we suggest that clinicians bear this diagnosis in mind when posed with a patient presenting with a tissue swelling with or without the presence of other symptoms of hypercalcaemia. This prevents the patient undergoing local surgical intervention by treating the primary endocrine abnormality.

- Patients with what appears to be a soft tumour of the mandible should have biochemical screening for hypercalcaemia
- In the first instance, if brown tumour is confirmed, the primary treatment should be parathyroidectomy
- Multidisciplinary management between otolaryngologists, endocrinologists, and radiologists including maxillofacial/head and neck surgeons should be undertaken
